# Diffusion tensor imaging and gray matter volumetry to evaluate cerebral remodeling processes after a pure motor stroke: a longitudinal study

**DOI:** 10.1007/s00415-024-12648-y

**Published:** 2024-09-03

**Authors:** Isabelle Loubinoux, Marie Lafuma, Julien Rigal, Nina Colitti, Jean-François Albucher, Nicolas Raposo, Mélanie Planton, Jean-Marc Olivot, François Chollet

**Affiliations:** 1 Toulouse NeuroImaging Center (ToNIC), Toulouse, France; 2 Neurology Department, Toulouse, France

**Keywords:** DTI MRI, Recovery of function, Diffusion tractography, Atrophy, Brain regeneration

## Abstract

**Background and objectives:**

Clinical factors are not sufficient to fix a prognosis of recovery after stroke. Pyramidal tract or alternate motor fiber (aMF: reticulo-, rubrospinal pathways and transcallosal fibers) integrity and remodeling processes assessable by diffusion tensor MRI (DTI) and voxel-based morphometry (VBM) may be of interest. The primary objective was to study longitudinal cortical brain changes using VBM and longitudinal corticospinal tract changes using DTI during the first 4 months after lacunar cerebral infarction. The second objective was to determine which changes were correlated to clinical improvement.

**Methods:**

Twenty-one patients with deep brain ischemic infarct with pure motor deficit (NIHSS score ≥ 2) were recruited at Purpan Hospital and included. Motor deficit was measured [Nine peg hole test (NPHT), dynamometer (DYN), Hand-Tapping Test (HTT)], and a 3T MRI scan (VBM and DTI) was performed during the acute and subacute phases.

**Results:**

White matter changes: corticospinal fractional anisotropy (FA_CST_) was significantly reduced at follow-up (approximately 4 months) on the lesion side. FAr (FA ratio in affected/unaffected hemispheres) in the corona radiata was correlated to the motor performance at the NPHT, DYN, and HTT at follow-up. The presence of aMFs was not associated with the extent of recovery. Grey matter changes: VBM showed significant increased cortical thickness in the ipsilesional premotor cortex at follow-up. VBM changes in the anterior cingulum positively correlated with improvement in motor measures between baseline and follow-up.

**Discussion:**

To our knowledge, this study is original because is a longitudinal study combining VBM and DTI during the first 4 months after stroke in a series of patients selected on pure motor deficit. Our data would suggest that good recovery relies on spared CST fibers, probably from the premotor cortex, rather than on the aMF in this group with mild motor deficit. The present study suggests that VBM and FA_CST_ could provide reliable biomarkers of post-stroke atrophy, reorganization, plasticity and recovery.

**ClinicalTrials.gov Identifier:**

NCT01862172, registered May 24, 2013

**Supplementary Information:**

The online version contains supplementary material available at 10.1007/s00415-024-12648-y.

## Introduction

As the leading cause of acquired adult disability, the third leading cause of death, stroke is a public health problem. Acute-phase therapeutic strategies such as thrombolysis and thrombectomy have improved functional prognosis. However, only 15% of stroke patients can benefit from these revascularization treatments and more than half of these treated patients have a Rankin score > 2 after 3 months [1]. Thus, motor deficit is the most common persistent clinical disability and affects approximately 80% of patients. At 6 months, 50% of patients over 65 years old have a persistent motor deficit [2]. The challenge is to better understand the mechanisms of neurological recovery and cerebral reorganization post-stroke. It is not known at this time whether the improvement in motor impairment is best explained by changes in structural connections or by functional connections of the motor system. A better knowledge of the mechanisms of cerebral plasticity can lead, in a second step, to therapeutic intervention studies. Reorganization and plastic changes underlying recovery also depend heavily on the phases of recovery [3] and homogeneously recruited populations will lead to more accurate findings. The study of focal lacunar infarcts is a first step towards understanding these plastic changes.

DTI is a magnetic resonance imaging technique for in vivo quantification of microstructural damage to white matter tracts following stroke. It assesses fractional anisotropy (FA) which is an index of Wallerian degeneration [4]. FA reflects the total amount of diffusion and is quite sensitive to a number of tissue properties, such as axonal ordering, axonal density, and degree of myelination, without being very specific to any one of them. It combines the contributions from the different sub-compartments of white matter into a single measure. Multiple prior studies on large infarcts have demonstrated a correlation between FA of the corticospinal tract (CST) and motor performance in stroke patients [5–7], including some longitudinal studies [7–9]. Other studies explore premotor or prefrontal pathways [10–14]. Studies more focused on lacunar infarcts corroborated these findings [10–12].

Reorganization and recovery probably depend also on remodeling in the cortical regions and indeed functional connectivity between cortical and subcortical networks. Voxel-based morphometry (VBM) has been developed by Ashburner and Friston in 2000 [15]. It evaluates brain structural abnormalities, including changes in gray matter, on T1-weighted MRI images. These structural changes are evaluated by comparing the assessment of local gray matter concentration between each voxel of images of two groups of subjects or on subjects explored longitudinally. Dang et al. [16], in a longitudinal MR study proved that changes in gray matter volumes, especially in specific motor-relevant brain regions occurring distal to the primary subcortical cerebral infarct, were associated with functional recovery after subcortical infarct. Furthermore, combining diffusion tensor imaging and VBM can better characterize natural evolution of cerebral remodeling processes after motor deficit.

To our knowledge, only three studies [17–19] have assessed the correlation between motor recovery using DTI and VBM together. These three studies included between 17 and 31 patients with various post-stroke follow-up times (between 3 months and several years) and no longitudinal follow-up. Besides, one study included ischemic and hemorrhagic strokes [19].

Here, we measure the longitudinal changes in FA_CST_ with DTI and remodeling in cortical regions with VBM and their relationship with motor recovery in a longitudinal study of post-stroke patients. Study of the corticospinal tract (FA_CST_, MD, AD, RD, mean, axial and radial diffusivities, respectively) was restricted to the tract emanating from the primary motor cortex. We hypothesized that corticospinal atrophy assessed by DTI and increased cortical thickness assessed by VBM would correlate with clinical recovery.

## Methods

### Subjects

Between February 2013 and July 2014, patients were recruited in this longitudinal study within the first 10 days after stroke via the stroke unit of Toulouse Hospital. Twenty-one patients (aged 18–90) with a pure motor deficit (NIHSS > 2) showed a first subcortical infarct proven by MRI. Cortical localization of the ischemic stroke, multiple stroke localizations, MRI contra-indication, coma, and history of psychiatric disease were exclusion criteria. Motor deficit was measured in the acute (first 10 days, baseline) phase and at follow-up (approximately 4 months after stroke onset) including the NIHSS, the Nine Peg Hole test (NPHT), the Hand-Tapping Test (HHT) and the dynamometer (DYN). This study was approved local Ethics Committee (Comité de Protection des Personnes Sud-Ouest et Outre-Mer I, n°12 483 03) and ANSM (Agence Nationale de Sécurité du Médicament et des produits de la santé, n°B121418-31) (ClinicalTrials.gov Identifier: NCT01862172, registered May 24, 2013).

### Imaging data acquisition

Imaging data were acquired at the same time points as clinical examination (first 10 days and approximately 4 months after stroke). A 3T Philips ACHIEVA MRI scanner and a 32-channel coil were used to acquire both diffusion-weighted imaging and high-resolution T1-weighted anatomic images. Fluid-attenuated inversion recovery (3D FLAIR; TR 8000 ms; TE 337 ms; inversion time 2400 ms; matrix 240 × 240 × 170; field of view (FOV) 240 × 240 × 170; voxel size 1 × 1 × 1, sagittal slices, duration time 6.16 min. For diffusion tensor imaging, 62 axial slices were obtained covering the whole brain with gradients (b = 1000 s/mm^2^) applied along 32 non-collinear directions with the following parameters: repetition time (TR): 7 s, echo time (TE) = 73 ms, FOV 224 × 224 × 124; voxel size = 2 × 2 × 2 mm, one B0, acquisition time: 9 min. Then, T1-weighted 3D magnetization prepared rapid gradient echo images (TR = 8,1 ms; TE = 3,7 ms; FOV = 220 × 132 × 170, voxel size = 1 × 1 × 1 mm) were also acquired in 4.20 min. To improve signal to noise ratio and reduced variability in acquisition [20], two acquisitions were made. Images of patient with a lesion in the right hemisphere were flipped, relative to the mid-sagittal plane, to the left hemisphere.

### Data processing

#### DTI

Diffusion-weighted images were analyzed using the diffusion spectrum imaging (DSI Studio) software (http://dsi-studio.labsolver.org). All data sets were corrected for eddy currents and head motion. With white matter Human Connectome Project atlas (HCP842 atlas), fiber tracking was based on an automatic fiber tracking function and a deterministic fiber tracking algorithm using a track recognition based on the tractography atlas [21]. The normalized diffusion images were superimposed on the MNI template for each subject to check normalization. Voxels at zero intensity were automatically eliminated. Fiber tracking was done by assigning regions as seeding region: primary motor cortex, ROIs: CR_sup_ (superior corona radiata), PLIC (posterior limb of the internal capsule), cerebral peduncle, and ending region: pontine crossing tract, and corticospinal tract. It was followed by the calculation of FA, MD, AD, and RD. The FA threshold was set to 0.2 and the angular threshold between 45 and 65°. DTI parameters were extracted for the corticospinal tract generated by the fiber tracking and the FA for three regions of interest (ROI) of the corticospinal tract: PLIC, corona radiata (CR) and pons. When the fibers generated by CST exploration drifted outside the true CST, correspondence with the corpus callosum, rubro- and reticulospinal (supplementary Fig. 1) tracts was also explored using the respective templates.

#### VBM

For VBM, we used the statistical Parametric toolbox (SPM 12) implemented on MATLAB and CAT12 (https://neuro-jena.github.io/cat/). VBM involves voxel-wise comparisons of local gray matter density or volume. The two T1 acquisitions acquired the same day were averaged (meanT1). For patients who had a lesion in the right hemisphere, their images were flipped, across the mid-sagittal plane, to the left hemisphere, for comparison purposes. Stroke lesions were masked out via manual tracing using MRIcron software and were not considered during the registration process. To do so, for each patient, we created a binary lesion mask depicting the lesion boundaries, using MRIcron software [22] (http://www.sph.sc.edu/comd/rorden/mricron/). The lesion was first identified using the T1 sequence, after which the lesion volume of interest (VOI) was drawn on each affected slice. The VOI was then smoothed using a 4-mm FWHM Gaussian filter with a 0.1% threshold [23]. A lesion-masked T1 was then created by merging the specific patient’s T1 and lesion VOI, using the ‘Imcalc’ function of SPM12 Toolbox (Wellcome Centre for Human Neuroimaging, London, UK; http://www.fil.ion.ucl.ac.uk/spm/) with the formula i1 ∗ .i2. CAT12 deals with lesions that have to be set to “0” in the images using the stroke lesion correction (SLC). These lesion areas are not used for segmentation or spatial registration, thus these preprocessing steps should be almost unaffected. Moreover, in the present stroke population, lesions are very small. For each patient, the structural MRI images underwent a procedure of a segmentation into gray matter, white matter and cerebrospinal fluid and a normalization onto the Montreal Neurological Institute (MNI) template. CAT uses geodesic shooting registration [24] with predefined templates. Finally, images were smoothed to suppress noise and effects due to residual differences in gyral anatomy, with a kernel of 6 × 6  × 6mm.

#### CST integrity

**Fig. 1:**
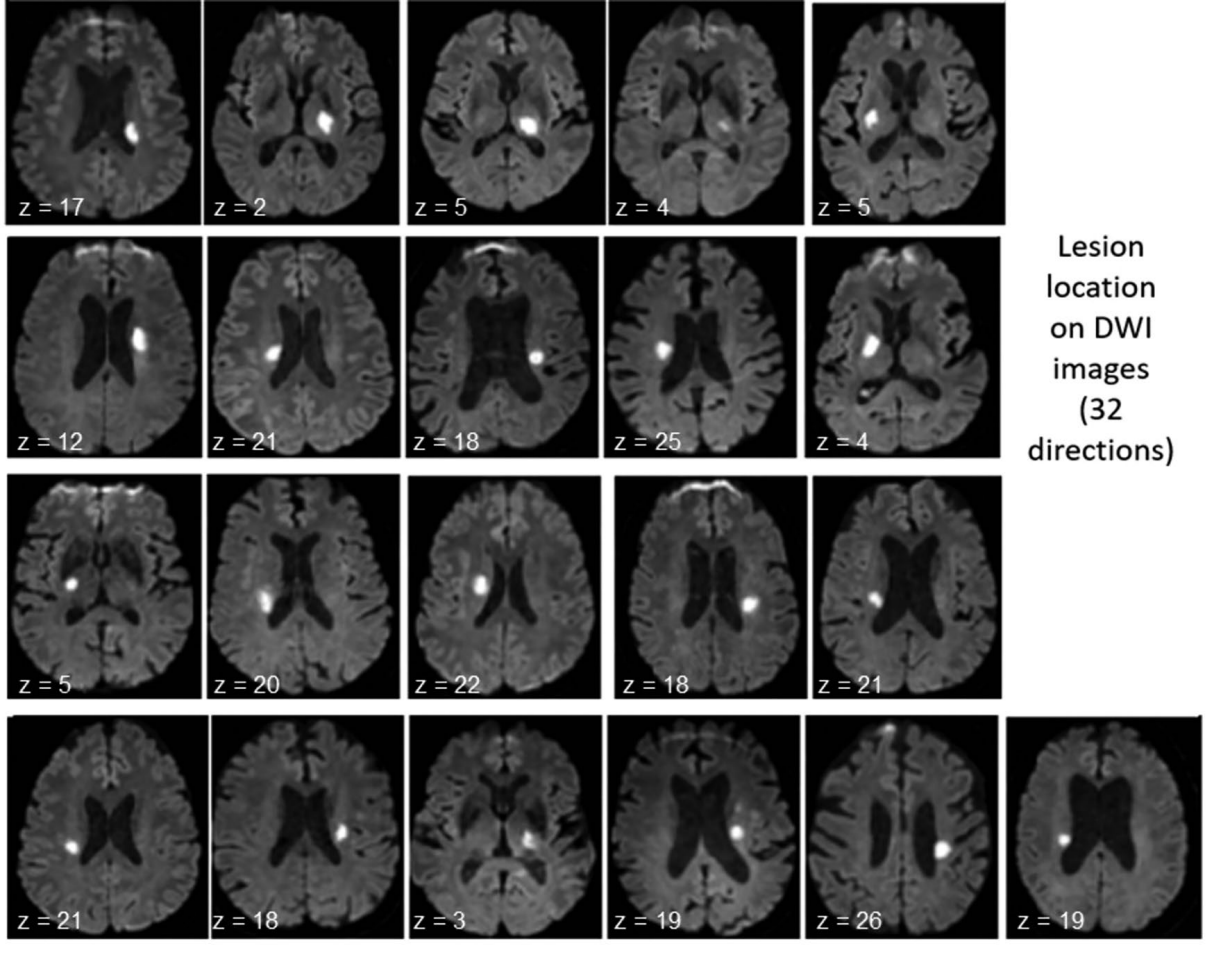
Lesion location on DWI images at baseline (*n* = 21 patients). z in mm above AC–PC plane

CST integrity was assessed according to the Lam Method [25] that was slightly modified because we thought that it was more pertinent to calculate an area of interrupted CST fibers instead of a volume. We chose the meanT1 axial slice perpendicular to the CST tract that showed the maximal overlap, as shown in Fig. 1. The percentage of CST lesion was calculated from the area of intersection between the lesion area and the CST area.

### Statistical analyses

#### DTI

Statistical analyses were performed using GraphPad Prism, and mean and SD were calculated. The threshold for statistical significance was set to *p* value < 0.05. The ratio of FA (FAr) was calculated by dividing the ipsilesional to the contralesional values. We first assessed the correlation between FAr on the three different ROIs and the motor tests at follow-up (approximately 4 months), using a linear regression model. To further probe the relationship between motor skill and microstructural status of white matter tracts, we classified patients in subgroups with mild or moderate deficit (threshold 30 s for the NPHT and 30 kg for the DYN) [26–28] and compared their FA using the parametric statistics, after checking that our data followed a normal law (with a Shapiro–Wilk test).

Alternative motor fibers: To assess the impact of aMF on clinical recovery, we initially separated patients into two groups. The first group was those from whom the tractography method found alternative pathways, either passing via the corpus callosum or via the rubro- and/or reticulospinal pathways and whatever the delay (baseline or follow-up). The second group that did not display alternative fibers, neither at baseline nor at follow-up. For each of these two groups, we calculated and compared the average clinical scores for the different tests at baseline and follow-up. We also calculated if there was a correlation between the presence of alternative motor fibers (aMF) and the progression of the recovery, i.e., difference between the score at follow-up and at baseline. Finally, we wanted to verify the impact of the presence of rubro- and/or reticulospinal pathways found by tractography at follow-up on motor recovery.

#### VBM

For longitudinal analysis using the SPM12 software, parametric tests were performed. We performed two analyses: first, whether there were any significant gray matter volume (GMV) changes across time, at baseline and follow-up for the whole patient cohort. For this analysis, a general univariate linear model and paired T-test was used under SPM12 to generate a statistical map comparing the baseline and follow-up T1 images. TIV (total intracranial volume) and age were included as covariates. When including age as covariate, no effect of age was found on the results. The threshold of significance set was *p* ≤ 0.001, cluster-level correction: clusters ≥ 100 voxels were considered as significant. The second analyses tested whether there were significant linear correlations between GMV and motor recovery scores. These second analyses were implemented as a simple regression model, with the GMV changes maps as a dependent variable, and the motor recovery scores as an independent variable (*p* < 0.05).

## Results

Demographic and clinical characteristics are shown in Table 1 and Fig. 1. All patients underwent clinical and MRI evaluation at baseline (4.4 days ± 2.7 from stroke onset) and at follow-up (approximately 4 months after baseline: 111 ± 22 days after baseline). None patient was lost at follow-up. At inclusion, no patient had an mRS of zero, 10 patients had an mRS of one, 6 had an mRS of two, and 5 had an mRS of four. At follow-up, 5 patients had an mRS of zero, 13 had an mRS of one, and 3 had an mRS of two. All patients improved in mRS, with the exception of one patient whose mRS worsened from 1 to 2. Mean lesional volume was 4.2 ± 1.9 ml. The NIHSS score was 2.80 ± 1.47 at baseline and 0.26 ± 0.56 at follow-up. Spontaneous motor recovery occurred at follow-up and 79% of the patient had a NIHSS score of 0 at follow-up. All patient received standard rehabilitation, which in France corresponds to 45 min-2 h/day for mild-to-moderate impaired patients.

**Table 1:** Clinical and demographics characteristics of the 21 patients. Baseline, i.e., at a mean of 4.42 days post-stroke; follow-up: at a mean of 116 days post-stroke

Demographic and clinical characteristics	Patients (n = 21)
Age	70 ± 10 [49–87]
Sex (female)	7/21 (33%)
Baseline NIHSS mean; SD; min/max	2.80 ± 1.47 [2–8]
Follow-up NIHSS; mean; SD; min/max	0.26 ± 0.56 [0–2]
NIHSS = 0 at follow-up	79%
mRS at baseline; mean; SD; min/max	2.0 ± 1.22 [1–4]
mRS at follow-up; mean; SD; min/max	0.95 ± 0.52 [0–2]
Lesion location Corona radiata PLIC Corona radiata/PLIC PLIC-thalamus PLIC-putamen-caudate	11 5 2 2 1
Lesion side	11 right/10 left
Mean lesional volume (mL) SD	4.2 ± 1.9
Mean % of damaged CST; SD; min, max	23.3% ± 16.0 [0–57]

### DTI analyses

Supplementary Table 1 shows the evolution of FA for the ipsi- and contralesional CST from the baseline and at follow-up. We calculated the evolution for three different parameters: meanFA for the CST and FA for two ROIs, posterior limb of the internal capsule (PLIC) and corona radiata (CR).

#### CST

There were no changes in axial, radial or mean diffusivity for the CST (Supplementary Table 2).

##### MeanFA of the entire CST

**Fig. 2:**
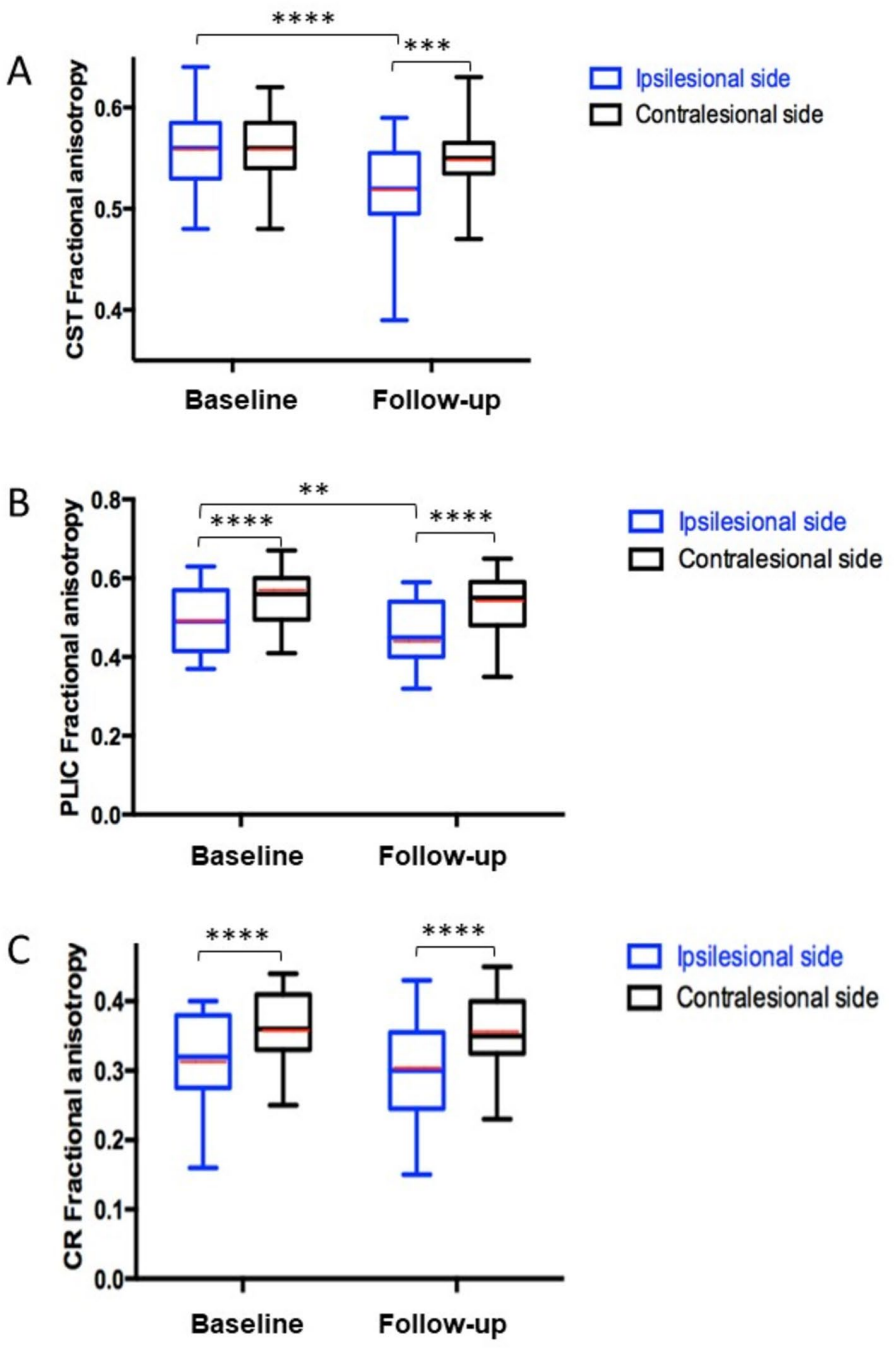
FA results. Graph of the evolution of mean FA between ipsi- and contralesional sides from the acute to the subacute stages for the entire CST (**A**), the PLIC (**B**), and the CR (**C**). Median, mean (red line), first and third quartiles, minima and maxima. *: *p* < 0.05; **: *p* < 0.005; ***: *p* < 0.001; ****: *p* < 0.0001

The ipsilesional meanFA_CST_ was not affected at baseline, whereas it was significantly decreased at follow-up compared to the contralesional side (Supplementary Table 1; Fig. 2).

##### FA of the posterior limb of the internal capsule

At baseline and follow-up, there was a statistical difference between the ipsilesional and contralesional side (Supplementary Table 1; Fig. 2). The ipsilesional FA was significantly lower between baseline and follow-up.

##### FA of the corona *radiata* tract

At baseline and follow-up, there was statistical difference between the ipsilesional and contralesional sides (Supplementary Table 1, Fig. 2).

#### Relation to the neurological motor deficit at follow-up

**Fig. 3:**
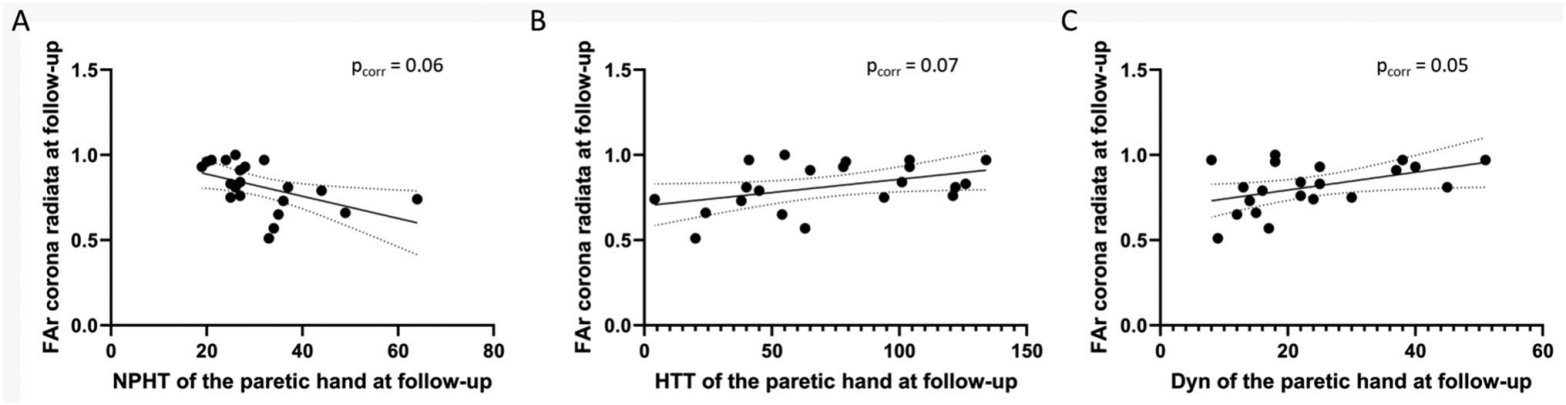
Correlation between MRI DTI parameters and motor test scores. Correlation of the FAr (ratio ipsi/contralesional FA) at follow-up in the ROI corona radiata and scores at the NPHT (**A**), HTT (**B**), and DYN (**C**) for the paretic side at follow-up. Pearson test corrected for multiple comparisons (Bonferroni–Holm correction) [29], respectively, *p* = 0.06, r^2^ = 0.25, *p* = 0.07, r^2^ = 0.18, *p* = 0.05, r^2^ = 0.20; 95% confidence interval

We correlated the FAr at follow-up (approximately 4 months) in the corona radiata ROI and the behavioural tests at follow-up with a regression analysis that showed a trend toward better function associated with a ratio closer to normal (value of one) (Fig. 3A, B, C).

Then, patients were categorized into two subgroups, according to their deficit (mild or moderate) at follow-up and according to previous proposed cutoff [27, 28]. For the NPHT, patients were considered having a moderate deficit if the test was performed in more than 29 s (*n* = 9), and having a mild deficit if the test was performed in less than 30 s (*n* = 12). For the dynamometer, a patient with a score less than 30 kg considered as moderately impaired and over 29 kg was considered as mildly impaired. For each of these two groups, we calculated the average of the FA on each of the 3 regions of interest: posterior limb of the internal capsule, corona radiata and pons (Suppl Table 3). For the NPHT measured at follow-up, the group with most severe initial deficit showed significant decreased FA in posterior limb of internal capsule and corona radiata (Suppl Table 3). For the dynamometer at follow-up, there was a significant group effect but post hoc analyses were not significant due to the low sample size in one group.

We calculated the percentage of CST lesion to assess CST integrity. We did not find any correlation, suggesting that CST integrity was not the only factor explaining motor deficit.

#### Alternative pathways

There is no statistically significant difference on the motor measures at any time between the two groups (aMF and no aMF) whatever the delay and whatever the test (Suppl Table 4).

### VBM analyses

**Fig. 4:**
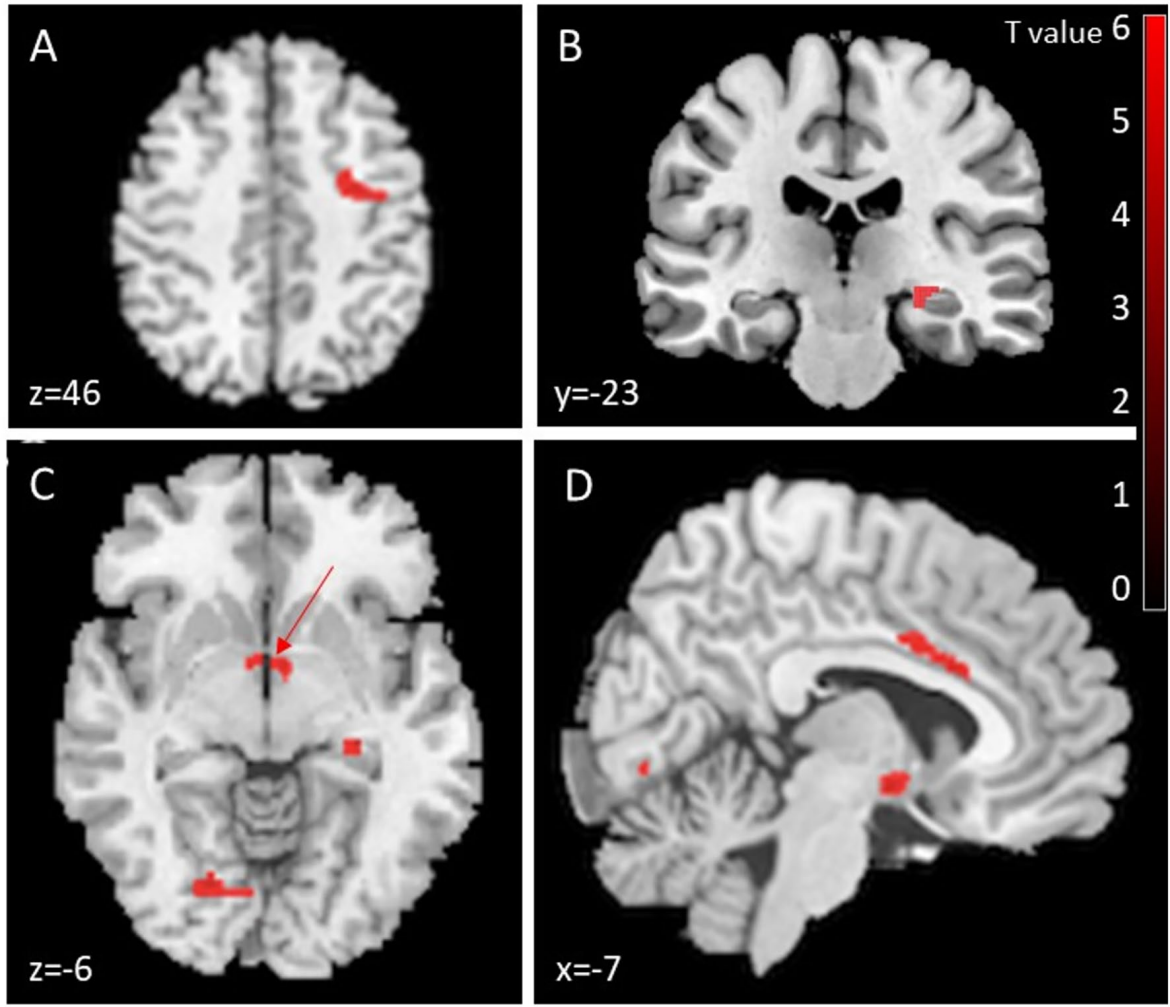
VBM results. VBM MRI axial, coronal and sagittal slices showing in red increased GM in the premotor cortex (**A**), the ipsilesional hippocampus (**B**), bilateral fornix (red arrow), lingual gyrus (**C**) and cingular anterior cortex (**D**) between baseline and follow-up (*p* ≤ 0.001, cluster-level correction: clusters ≥ 100 voxels)

Voxel-wise paired t-tests on the gray matter (GM) between the acute and subacute stages of stroke demonstrated significant increase in GM in the ipsilesional premotor cortex, hippocampus and fornix, lingual gyrus and cingular anterior cortex (Fig. 4; Table 2). No decrease in GM suggesting atrophy was evidenced.

**Table 2:** Brain regions exhibiting increased GM at follow-up (approximately 4 months) (*p* < 0.001). MNI: Montreal Neurological Institute

Brain region	MNI coordinates of maximum (X, Y, Z)	Cluster size (voxels)
Increased gray matter		
Ipsilesional premotor cortex	(42;− 4.5; 42)	337
Ipsilesional hippocampus Bilateral fornix	(27; − 25.5; − 7.5) (− 3; − 3; − 1)	179
Lingual gyrus	(− 19.5; − 73.5; 6)	162
Cingular anterior cortex	(− 6; 6; 31.5)	125

#### Correlations between the changes in GM and the clinical variables

**Fig. 5:**
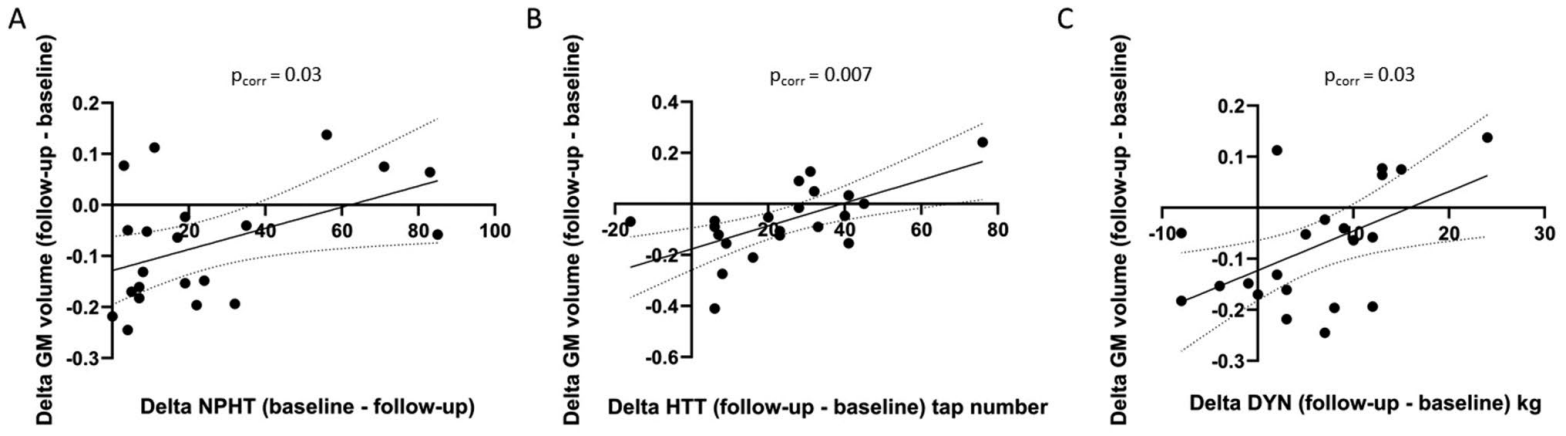
Correlation between MRI VBM parameters and motor score changes. Correlation between the change in gray matter volume in the contralesional anterior cingulum and the improvement at the NPHT (**A**), HTT (**B**), and DYN (**C**) between baseline and follow-up. (Pearson test corrected for multiple comparisons, *p* = 0.03, r^2^ = 0.22, *p* = 0.007, r^2^ = 0.39; *p* 0.03, r^2^ = 0.27; 95% confidence interval; x = − 9; y = 21; z = 27)

GMV changes and the changes in the motor measures between follow-up and baseline were positively correlated in the anterior cingulum for the NPHT, HTT and DYN (Fig. 5A, B, C, p < 0.05, corrected).

## Discussion

As far as we know, this is the first human study looking at both DTI and VBM changes longitudinally and in a very selected population of stroke patients with pure motor deficits.

For the evolution of FA, we compared ipsi- and contralesional CST at the acute and subacute phase post-stroke. The DTI analysis was focused on the CST originating in the primary motor cortex. In the acute phase (first 10 days), there was no difference between ipsi- and contralesional CST. This result is consistent to previous studies [7, 8, 16–19, 25, 30–32]. Puig et al. found absolute and relative FA decreases at 30 days after stroke, but not at 12 h or 3 days after stroke [7], whereas Doughty et al. found a subtle but significant changes in FA in cerebral peduncles, however, with larger lesions [8].

However, when we calculate the FA in a ROI involving the lesion (e.g., posterior limb of internal capsule) or above (corona radiata), in the acute phase, FA was statistically lower in the affected side, meaning that the Wallerian degeneration had started but only around the lesion. This change was detectable despite small lesions. At follow-up, the FA was statistically lower in the ipsilesional than in the contralesional CST, because retrograde Wallerian degeneration had occurred along the pyramidal tract. We showed a significant correlation between FA and motor function of the affected side (NPHT, HTT, and DYN) and could classify patients according to the severity of the deficit.

FA values represent the degree of directionality of microstructures (e.g., axons, myelin and microtubules). Reduced FA values appear to be related to the disintegration of fibers and Wallerian degeneration. These results are in agreement with the previous studies that evaluate the relationship between the CST status and motor function in stroke patients [33]. We found that the FAr of CST accounts for 18–25% of the variability of the motor status, which is relatively lower than previous reports. One of the reason is that, at follow-up, most patients have recovered well and homogeneously (Table 1). It, therefore, appears more difficult to establish correlations.

Alternate motor fibers (aMF) which includes reticulo-, rubrospinal pathways and transcallosal fibers may also be important for recovery [10, 13, 34]. However, their post-stroke role, benefit or harmful, is not fully understood, and may vary, depending on the post-stroke stage [35]. In DTI studies, it seems that the presence of aMF in the chronic post-stroke phase is beneficial [30]. Nevertheless, there is no longitudinal DTI studies that evaluate the evolution of aMF during acute and subacute stages of stroke and their impact on clinical recovery.

In our study, we did not find any correlations between the probabilistic presence of alternate motor fibers detected by tractography and motor recovery, whatever the post-stroke stage (acute or subacute). Among this group of patients recovering well, it could demonstrate different plasticity processes, proving effective individual recovery strategies. Lindenberg et al. [30] in a 35 chronic stroke patient study found poor recovery when both tracts (CST and aMF) were affected, but moderate or better recovery when the aMF tract was unaffected. Karbasforoushan et al. measured the FA of CST and alternate motor tracts in the spine and found that motor recovery relied on the contralesional medial reticulospinal tract in severely impaired patients [36]. However, our study shows a small or punctual involvement of alternate motor tracts in mildly impaired patients.

With VBM, we found significant increased GM volumes in areas distant from the primary lesion site between the acute and subacute stages post-stroke. Four sites presented increased cortical thickness at follow-up. The ipsilesional premotor cortex was the larger location. It corresponds to Brodmann’s area 6, which is directly connected to infarcted areas in the present study. Indeed, the premotor cortex emits 20% of the fibers constituting the CST and also sends efferences to the primary motor cortex. The upper limb is represented by a larger number of neurons than in the primary motor cortex [37]. Abela et al. [38], in a prospective study of 28 patients, also found increased cortical thickness of the premotor ipsilesional cortex at 3 and 9 months post-infarction. No effects related to the injured hemisphere were found in the regions described in the present VBM study in subcortical patients [39]. Thus, the flipping procedure we applied does not challenge our results.

We can wonder what is the link between DTI and VBM changes and whether the Wallerian degeneration of the CST has a consequence on the increased GM in the ipsilesional premotor cortex. However, no correlation was found. It can be hypothesized that this increased cortical thickness of the ipsilesional premotor cortex reflects both restorative and compensatory phenomenons for the hand involving i. redundancy of the hand representation and more involvement of CST premotor fibers and II. maybe a vicariance process with the take of a new role of premotor neurons in driving motor command [40].

As for the hippocampus, it is consistent with different previous studies. Fan et al. in 10 patients had also found increased cortical thickness of the hippocampus that correlated with clinical recovery [41]. Yu et al. found increased cortical thickness of the hippocampus at 6 months in 12 patients [42]. Finally, Gauthier emphasized the contribution of the structural plasticity of the hippocampus to the therapy-induced recovery of motor function in stroke patients after constraint-induced movement therapy [43]. An increased cortical thickness of fornix fibers connecting the mammillary bodies to the hippocampus is in agreement with these results. Eriksson et al., in 1998 [44], showed that human hippocampus retains its ability to generate neurons throughout life. Jin et al. showed that there were small capacities of neurogenesis in the periventricular zone and hippocampus post-stroke [45]. We can wonder if the recruitment of cognitive resources is beneficial on motor recovery. This hippocampal increased cortical thickness was not associated with an improvement in cognitive performance nor in mnesic capacities since the patients were not impaired but may be due to a relearning of the motor functions.

The lingual gyrus has a role in the visual perception of movement. Visual analysis of the movement of the healthy limb could allow recovery and thus lead to increased cortical thickness of the lingual gyrus. Two studies also found increased cortical thickness of this gyrus [41, 42]. The two latter localizations suggest that non-motor function such as cognition and vision are recruited to facilitate motor recovery after stroke since ‘spatiality’ is an important features of movements.

The anterior cingulate cortex has direct motor connections to the different cortical regions involved in motor control: the premotor cortex and the supplementary motor area. It corresponds to Brodmann area 24. This increased cortical thickness would, therefore, correspond to an adaptive plasticity and a direct compensating motor phenomenon since it was correlated to improved recovery.

In the present longitudinal MRI study, even if lesions were relatively small, some cortical brain regions showed significant changes in volume sometimes with a statistical correlation with motor recovery. This reorganization would, therefore, be a restorative phenomenon. The hypothesis underlying cortical thickness modifications are derived from pre-clinical histological studies in rats [40, 46–51]. Increased cortical thickness is possibly linked to a neurogenesis. It could also be a dendritic sprouting or synaptogenesis. Neurogenesis abilities have been described by Eriksson and Jin in post-stroke patients [44, 45].

Our study has some limitations: it applies only to patients having a mild or moderate deficit and a good recovery at 4 months making difficult to establish correlations between clinical outcomes and microstructural modifications. However, we found a correlation with the anterior cingulum. The group was homogeneous with good recovery and only 23.3% loss of CST fibers suggesting that enough direct fibers and minor reorganization can sustain recovery without the intervention of alternate motor fibers (40). Another limitation was that we explore the CST emanating from M1 only and did not examine the part originating from the premotor cortex. However, since we show that in this group of patients with very small lesions, reorganization takes place in the premotor cortex, pulling the latter with the primary cortex could have put together opposite changes, degenerating and regenerating processes. The strengths of our study are a longitudinal follow-up at two different time points post-stroke (acute and subacute) and a homogeneous group recruited on a pure motor deficit.

## Conclusion

In the present study, we confirmed that motor outcome is highly dependent on lesion location and particularly on CST microstructural status. We were able to identify post-stroke adaptive processes with two different neuroimaging techniques that showed complementary results. DTI tractography demonstrated substantial Wallerian degeneration, and suggested the presence of alternative circuits in some patients via the corpus callosum or the rubro- and reticulospinal pathways, which were not evidenced, however, to be associated with motor recovery in this study. This comforted us to interpret the VBM increased cortical thickness of the premotor cortex, anterior cingulum and hippocampus as substrate of a successful strategy of recovery, mainly implicating premotor CST fibers. Indeed, DTI and VBM give potential predictive biomarkers of post-stroke recovery. They make it possible to evaluate both the anatomical and functional reorganization. A better knowledge of the mechanisms of brain plasticity may lead to more precise therapeutic interventions.

## Supplementary Information

Below is the link to the electronic supplementary material.

Supplementary file1 (PPTX 207 kb)

## Data Availability

The data that support the findings of this study are available from the corresponding author upon reasonable request.
