# Supplementary material for: Diffusion tensor imaging and gray matter volumetry to evaluate cerebral remodeling processes after a pure motor stroke: a longitudinal study
Source: J Neurol. 2024 Sep 3;271(10):6876–87. doi: 10.1007/s00415-024-12648-y (PMC11447101; doi:10.1007/s00415-024-12648-y)
Supplement: Supplementary file 1 — Supplementary file1 (PPTX 207 kb) [file 415_2024_12648_MOESM1_ESM.pptx]

## Slide 1
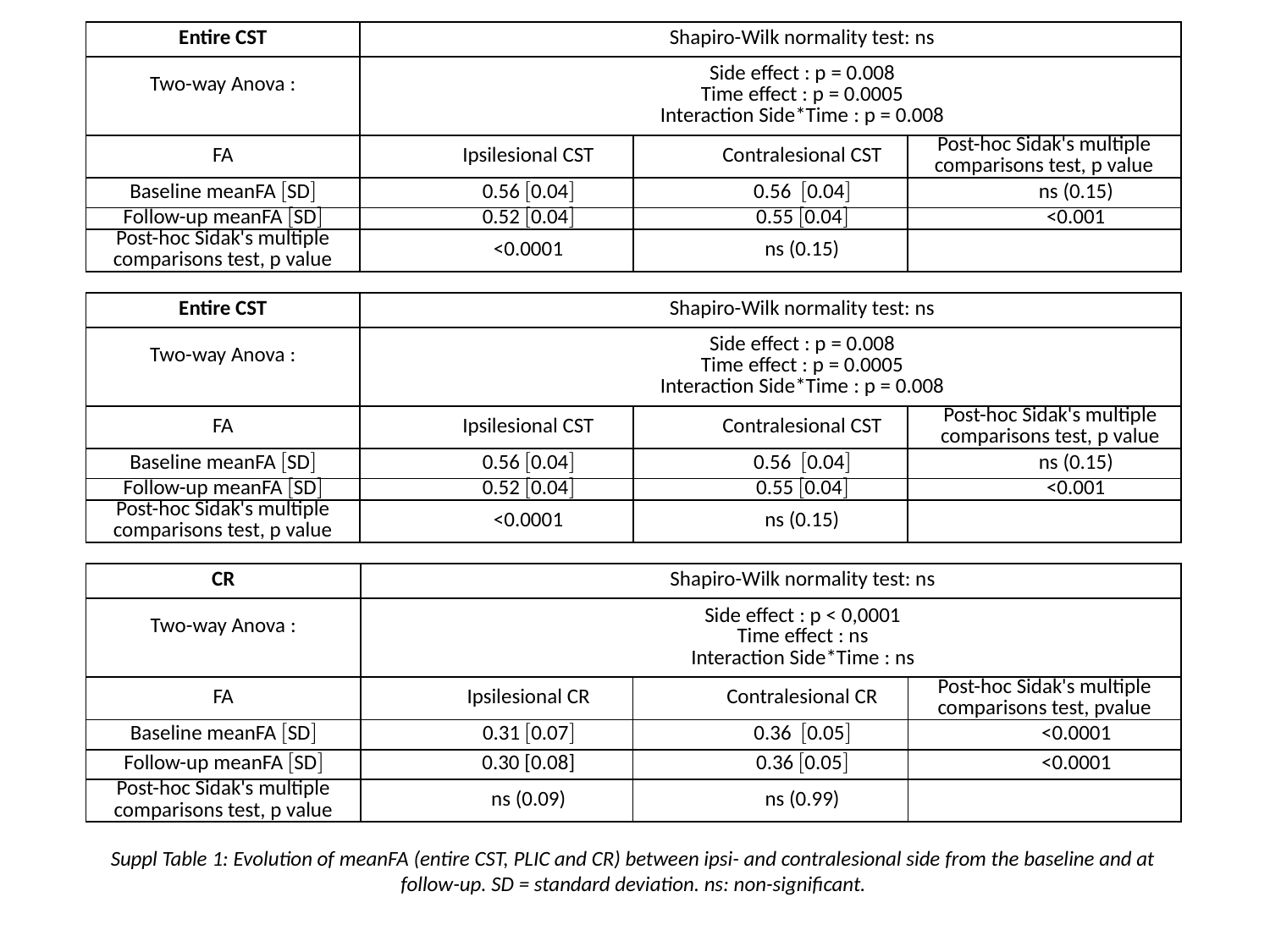

| Entire CST | Shapiro-Wilk normality test: ns | | |
| --- | --- | --- | --- |
| Two-way Anova : | Side effect : p = 0.008 Time effect : p = 0.0005 Interaction Side\*Time : p = 0.008 | | |
| FA | Ipsilesional CST | Contralesional CST | Post-hoc Sidak's multiple comparisons test, p value |
| Baseline meanFA SD | 0.56 0.04 | 0.56 0.04 | ns (0.15) |
| Follow-up meanFA SD | 0.52 0.04 | 0.55 0.04 | <0.001 |
| Post-hoc Sidak's multiple comparisons test, p value | <0.0001 | ns (0.15) | |
| Entire CST | Shapiro-Wilk normality test: ns | | |
| --- | --- | --- | --- |
| Two-way Anova : | Side effect : p = 0.008 Time effect : p = 0.0005 Interaction Side\*Time : p = 0.008 | | |
| FA | Ipsilesional CST | Contralesional CST | Post-hoc Sidak's multiple comparisons test, p value |
| Baseline meanFA SD | 0.56 0.04 | 0.56 0.04 | ns (0.15) |
| Follow-up meanFA SD | 0.52 0.04 | 0.55 0.04 | <0.001 |
| Post-hoc Sidak's multiple comparisons test, p value | <0.0001 | ns (0.15) | |
| CR | Shapiro-Wilk normality test: ns | | |
| --- | --- | --- | --- |
| Two-way Anova : | Side effect : p < 0,0001 Time effect : ns Interaction Side\*Time : ns | | |
| FA | Ipsilesional CR | Contralesional CR | Post-hoc Sidak's multiple comparisons test, pvalue |
| Baseline meanFA SD | 0.31 0.07 | 0.36 0.05 | <0.0001 |
| Follow-up meanFA SD | 0.30 [0.08] | 0.36 0.05 | <0.0001 |
| Post-hoc Sidak's multiple comparisons test, p value | ns (0.09) | ns (0.99) | |
Suppl Table 1: Evolution of meanFA (entire CST, PLIC and CR) between ipsi- and contralesional side from the baseline and at follow-up. SD = standard deviation. ns: non-significant.

## Slide 2
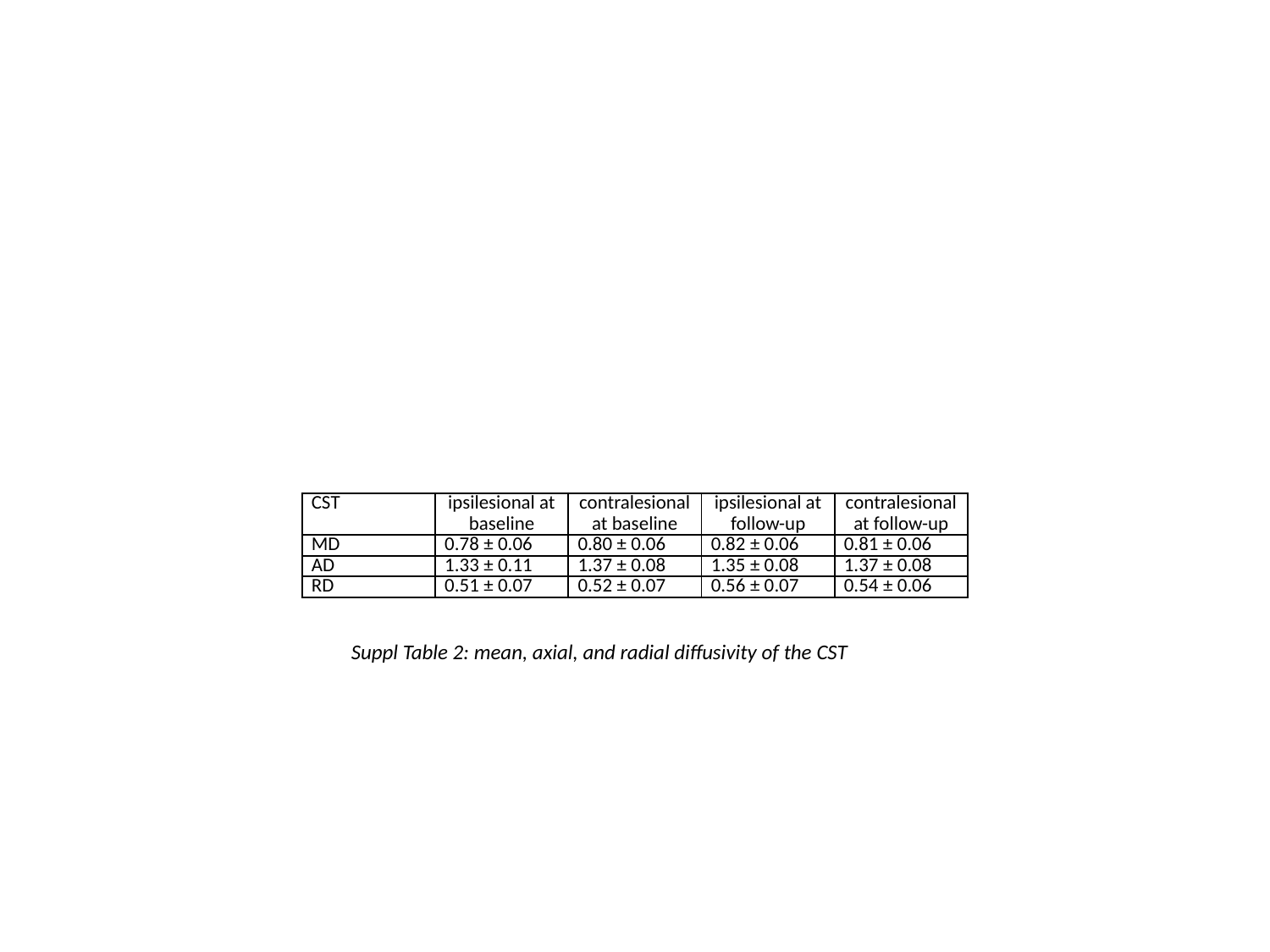

| CST | ipsilesional at baseline | contralesional at baseline | ipsilesional at follow-up | contralesional at follow-up |
| --- | --- | --- | --- | --- |
| MD | 0.78 ± 0.06 | 0.80 ± 0.06 | 0.82 ± 0.06 | 0.81 ± 0.06 |
| AD | 1.33 ± 0.11 | 1.37 ± 0.08 | 1.35 ± 0.08 | 1.37 ± 0.08 |
| RD | 0.51 ± 0.07 | 0.52 ± 0.07 | 0.56 ± 0.07 | 0.54 ± 0.06 |
Suppl Table 2: mean, axial, and radial diffusivity of the CST

## Slide 3
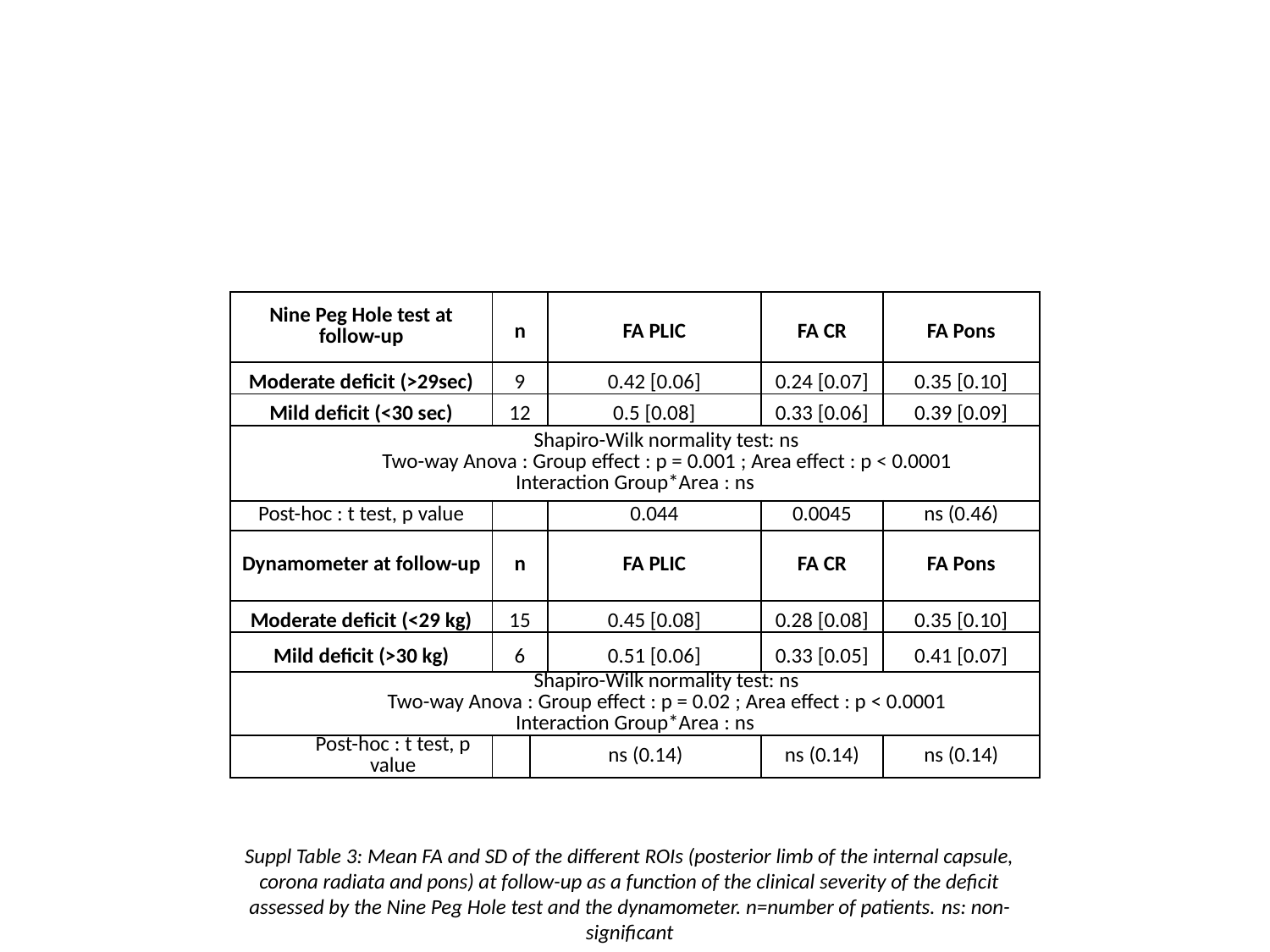

| Nine Peg Hole test at follow-up | n | | FA PLIC | FA CR | FA Pons |
| --- | --- | --- | --- | --- | --- |
| Moderate deficit (>29sec) | 9 | | 0.42 [0.06] | 0.24 [0.07] | 0.35 [0.10] |
| Mild deficit (<30 sec) | 12 | | 0.5 [0.08] | 0.33 [0.06] | 0.39 [0.09] |
| Shapiro-Wilk normality test: ns Two-way Anova : Group effect : p = 0.001 ; Area effect : p < 0.0001 Interaction Group\*Area : ns | | | | | |
| Post-hoc : t test, p value | | | 0.044 | 0.0045 | ns (0.46) |
| Dynamometer at follow-up | n | | FA PLIC | FA CR | FA Pons |
| Moderate deficit (<29 kg) | 15 | | 0.45 [0.08] | 0.28 [0.08] | 0.35 [0.10] |
| Mild deficit (>30 kg) | 6 | | 0.51 [0.06] | 0.33 [0.05] | 0.41 [0.07] |
| Shapiro-Wilk normality test: ns Two-way Anova : Group effect : p = 0.02 ; Area effect : p < 0.0001 Interaction Group\*Area : ns | | | | | |
| Post-hoc : t test, p value | | ns (0.14) | | ns (0.14) | ns (0.14) |
Suppl Table 3: Mean FA and SD of the different ROIs (posterior limb of the internal capsule, corona radiata and pons) at follow-up as a function of the clinical severity of the deficit assessed by the Nine Peg Hole test and the dynamometer. n=number of patients. ns: non-significant

## Slide 4
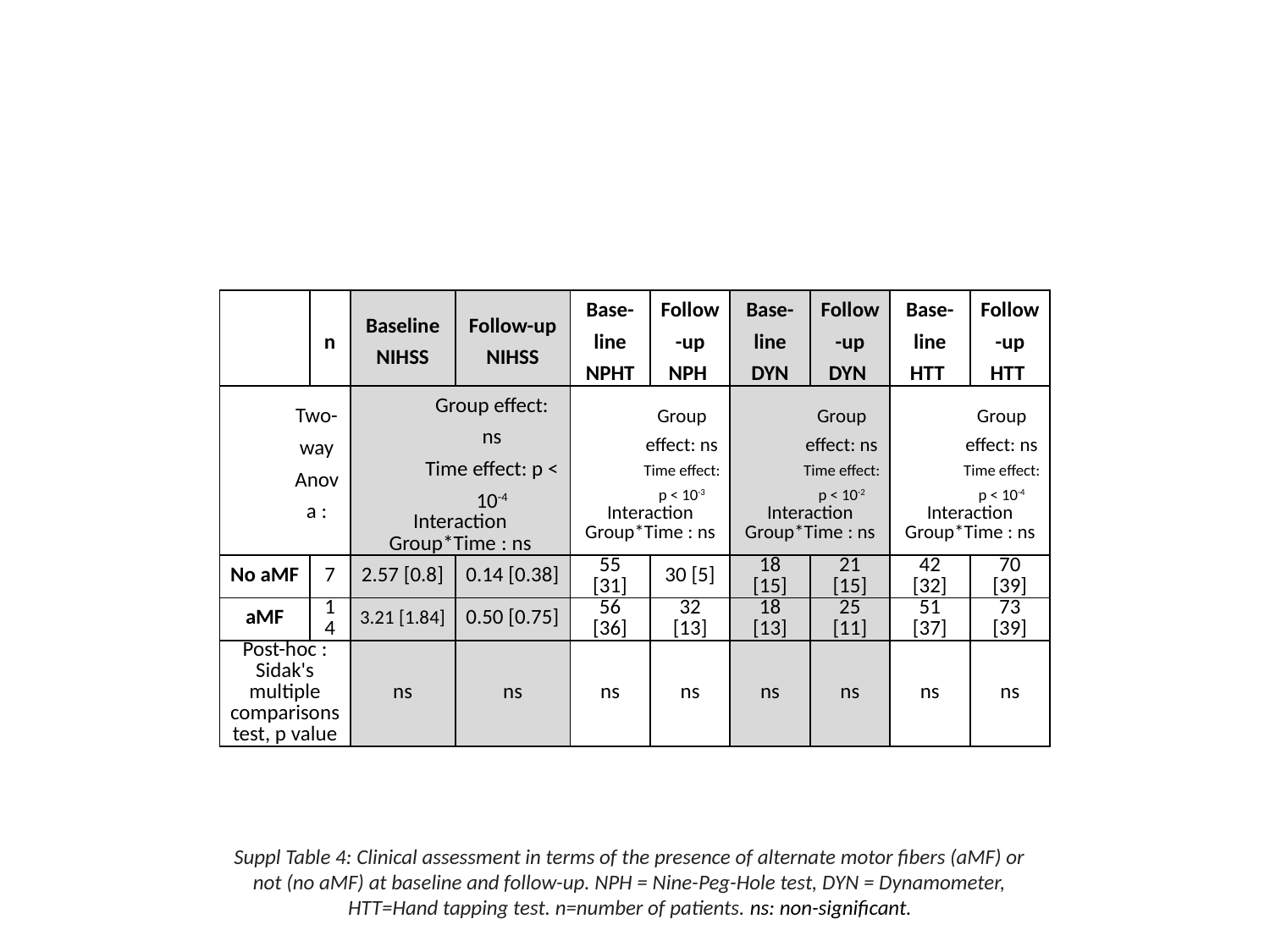

| | n | BaselineNIHSS | Follow-up NIHSS | Base-line NPHT | Follow-up NPH | Base-line DYN | Follow-up DYN | Base-line HTT | Follow-up HTT |
| --- | --- | --- | --- | --- | --- | --- | --- | --- | --- |
| Two-way Anova : | | Group effect: ns Time effect: p < 10-4 Interaction Group\*Time : ns | | Group effect: ns Time effect: p < 10-3 Interaction Group\*Time : ns | | Group effect: ns Time effect: p < 10-2 Interaction Group\*Time : ns | | Group effect: ns Time effect: p < 10-4 Interaction Group\*Time : ns | |
| No aMF | 7 | 2.57 [0.8] | 0.14 [0.38] | 55 [31] | 30 [5] | 18 [15] | 21 [15] | 42 [32] | 70 [39] |
| aMF | 14 | 3.21 [1.84] | 0.50 [0.75] | 56 [36] | 32 [13] | 18 [13] | 25 [11] | 51 [37] | 73 [39] |
| Post-hoc : Sidak's multiple comparisons test, p value | | ns | ns | ns | ns | ns | ns | ns | ns |
Suppl Table 4: Clinical assessment in terms of the presence of alternate motor fibers (aMF) or not (no aMF) at baseline and follow-up. NPH = Nine-Peg-Hole test, DYN = Dynamometer, HTT=Hand tapping test. n=number of patients. ns: non-significant.

## Slide 5
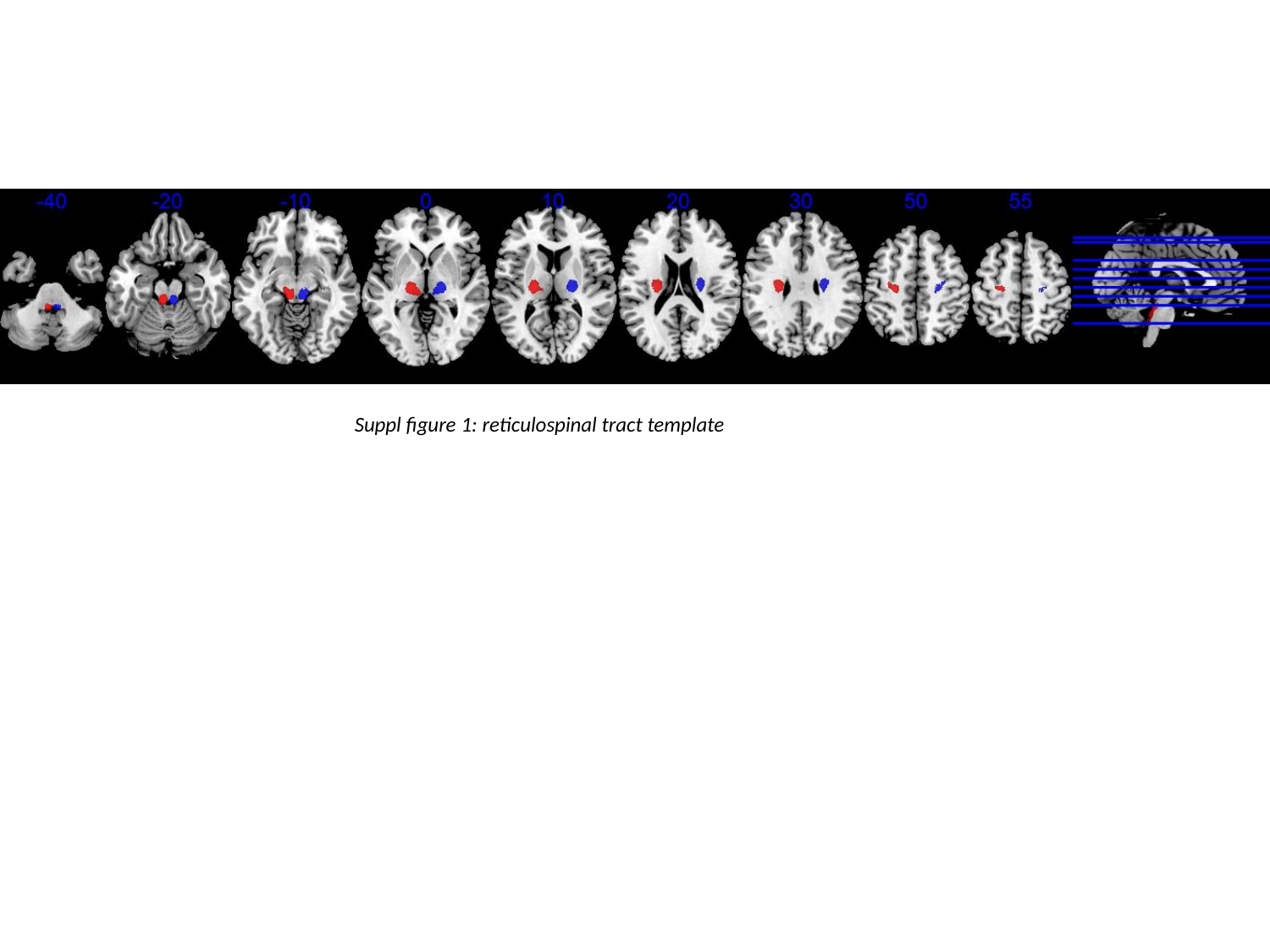

Suppl figure 1: reticulospinal tract template
